# Phylogenetics beyond biology

**DOI:** 10.1007/s12064-018-0264-7

**Published:** 2018-06-21

**Authors:** Nancy Retzlaff, Peter F. Stadler

**Affiliations:** 1 Max-Planck Institute for Mathematics in the Sciences, Inselstraße 22, 04103 Leipzig, Germany; 2 Interdisciplinary Center of Bioinformatics, University of Leipzig, Härtelstrasse 16-18, 04107 Leipzig, Germany; 3 Bioinformatics Group, Department of Computer Science and Interdisciplinary Center of Bioinformatics, University of Leipzig, Härtelstrasse 16-18, 04107 Leipzig, Germany; 4 Fraunhofer Institut für Zelltherapie und Immunologie – IZI, Perlickstraße 1, 04103 Leipzig, Germany; 5 Institute for Theoretical Chemistry, University of Vienna, Währingerstraße 17, 1090 Vienna, Austria; 6 Center for Non-coding RNA in Technology and Health, University of Copenhagen, Grønnegårdsvej 3, 1870 Frederiksberg C, Denmark; 7 Santa Fe Institute, 1399 Hyde Park Rd., Santa Fe, NM 87501 USA

**Keywords:** Cultural evolution, Phylogenetic tree, Additive metric, Metric-preserving functions

## Abstract

Evolutionary processes have been described not only in biology but also for a wide range of human cultural activities including languages and law. In contrast to the evolution of DNA or protein sequences, the detailed mechanisms giving rise to the observed evolution-like processes are not or only partially known. The absence of a mechanistic model of evolution implies that it remains unknown how the distances between different taxa have to be quantified. Considering distortions of metric distances, we first show that poor choices of the distance measure can lead to incorrect phylogenetic trees. Based on the well-known fact that phylogenetic inference requires additive metrics, we then show that the correct phylogeny can be computed from a distance matrix ${\mathbf {D}}$ if there is a monotonic, subadditive function $\zeta$ such that $\zeta ^{-1}({\mathbf {D}})$ is additive. The required metric-preserving transformation $\zeta$ can be computed as the solution of an optimization problem. This result shows that the problem of phylogeny reconstruction is well defined even if a detailed mechanistic model of the evolutionary process remains elusive.

## Introduction

At the most abstract level, evolution can be seen as a consequence of the generation of variation and selection. Since selection acts to remove entities from the system, it will eventually “die out” unless counteracted by some form of reproduction. Sustained evolution thus necessarily operates on populations of entities. The history of an evolutionary process can be recorded in the form of a directed graph: Dress et al. (2010b) considered the set $Y$ comprising “all organisms that ever lived on earth” arranged into a graph $G$ with arcs (directed edges) connecting to nodes $u$ and $v$ whenever $u$ was a “parent” of $v$, defined in a rather loose sense as having contributed directly to the genetic make-up of $v$. These arcs encode not only father and mother in sexually reproducing populations, but also horizontal gene transfer, hybridization, the incorporation of retroviruses into the genome, etc. Since arcs encode ancestry, $G$ is acyclic.

The very same construction applies to many other systems that are perceived as evolutionary. For example, in the evolution of languages one may consider the mutual influences of speakers or, even more fine grained, individual utterances as the basic entities (Croft 2000; Pagel 2009). The same is true for the transmission of cultural techniques, designs, and conventions (Mesoudi et al. 2006). Well-studied cases include the transmission of texts (Greg 1950), in particular manuscripts, and text reuse, i.e., the borrowing of parts of a corpus, with or without modifications, in the process of creating a new text, see, e.g., Seo and Croft (2008). Similarly, the revisions of the law as dissenting interpretations can be seen in this manner (Roe 1996). The common ground of these and presumably many other systems is that a limited set of entities at some point or interval in time “informs” limited sets of entities in their (usually immediate) future.

The key result of Dress et al. (2010b) is that several types of clusters on the subset $U\subset Y$ of organisms that are currently alive can be defined from the structure of the graph $G.$ Many of these form hierarchies and therefore define a tree. These clusters naturally take on the role of taxa, and the corresponding trees consequently are a meaningful representations of the phylogenetic relationships among these taxa. The same interpretation is meaningful, as we argued above, also for many—but presumably not all—aspects of human cultural endeavors. Notions of cultural evolution (see, e.g., Flannery (1972), Mesoudi et al. (2006)) are therefore more than a convenient metaphor. Instead, for a given system of interest, one has to ask whether or not the corresponding graph $G$ shares key features with the one obtained from conceptualizing biological evolution. There is no *a priori* reason to assume, for instance, that $G$ always gives rise to the tree-like abstraction that is at the heart of biological evolution. This is an inherently empirical question that needs to be answered for each “evolutionary” system under consideration. Human languages, for instance, are a prime example of an aspect of human activity that closely conforms to biological evolution.

The key point here is that a phylogenetic structure is an emergent phenomenon of the underlying evolutionary process; it requires that there exists a level of aggregation in *G* that produces clusters adhering to an (essentially) hierarchical structure. Although Dress et al. (2010b) provide a formal justification for phylogenetic reconstruction with their analysis of the graph $G$, their work does not attempt to provide a practical procedure to identify the relevant clusters, i.e., the taxa. After all, these are defined in terms of the graph $G$, which of course is not directly observable. In fact, usually not even the set $U$ of extant entities will be known completely, as we will have to be content with a subset of available data.

In general, neither the “true nature” of the elementary entities nor a complete description for each of them is available to us. Instead, we have to be content with *measured* representations. For instance, in molecular phylogenetics, it is customary to *represent* a taxon by a set of sequences (usually representing single copy protein coding genes) obtained from one or more individuals. Morphological approaches in phylogenetics use a list of characters such as features of bones or organs to represent a typical individual. The impact of the choice of representation on the results of phylogenetic reconstructions has long been recognized in morphological phylogenetics and has been the subject of a long-standing debate, see, e.g., Wiens (2001).

The fundamental assumption that is made in any type of similarity-based phylogenetic analysis is that similarity of representations reflects evolutionary relatedness, i.e., proximity in $G$, and therefore also makes it possible to identify the hierarchical cluster systems that are defined in terms of $G$. This is well established, of course, in the case of molecular phylogenetics, where a detailed model of sequence evolution is available (Jukes and Cantor 1969; Tavaré 1986; Arenas 2015). Similarly, permutation distances directly count genomic rearrangement events (Hannenhalli and Pevzner 1995). The connection is much less clear for morphological phylogenetics, where choice and even the concept of “character” is under debate, see, e.g., Wagner (2001), Wagner and Stadler (2003) for a formal discussion. In many cases, it seems difficult to construct a theory that links distance or similarity measures directly to an underlying evolutionary process. This is the case for instance in phylogenetic applications of distances between RNA secondary structures (Siebert and Backofen 2005) or the use of distance measures based on data compression (Cilibrasi and Vitanyi 2005; RajaRajeswari and Viswanadha Raju 2017).

Phylogenetic methods have also been employed in the humanities. Relationships among languages, for instance, can be captured by using cognates (i.e., words with a common origin) as characters, see, e.g., Gray et al. (2011), Holman and Wichmann (2017). Recently, sophisticated statistical approaches, that model, e.g., the importance of sound change, have been used to reconstruct language trees, see, e.g., Bhattacharya et al. (2018) for a recent overview. In stemmatics, differences between editions or manuscripts serve as characters from which the relationships, e.g., between the many different versions (O’Hara and Robinson 1993; Barbrook et al. 1998; Marmerola et al. 2016) can be reconstructed. Occasionally, material artefacts are considered. Tëmkin and Eldredge (2007) studied used phylogenetic methods to study the history of certain musical instruments. A broader perspective of phylogenetic approaches in cultural evolution is discussed, e.g., by Mesoudi et al. (2006), Steele et al. (2010) or Howe and Windram (2011).

It is a well-known fact in sequence analysis that not all (reasonable) distance measures lead to faithful reconstructions of phylogenies. It is a well-established practice, in fact, to correct for back-mutations, i.e., to transform raw counts of diverged sequence positions, i.e., the Hamming or Levenshtein distances, into distance measures that can be interpreted as numbers of evolutionary events or divergence times. Depending on the level of insights into the data, the simple Jukes–Cantor model (Jukes and Cantor 1969) or one of the many much more elaborate models (Tavaré 1986; Arenas 2015) is used for this purpose. In the field of alignment-free sequence analysis, on the other hand, the focus is on the efficient computation of dissimilarity measures, without overt concern of the measure’s connection to a dynamical model of evolution (Vinga and Almeida 2003). One has observed, however, the distance measures that do well in a phylogenetic context also correlate very well with model-based distances (Edgar 2004; Haubold et al. 2009; Leimeister and Morgenstern 2014). We suspect that this reflects the fact that a particular subclass of metrics, the so-called additive metrics, conveys complete phylogenetic information, see “Distance-based phylogenetics” section. We therefore make a strong assumption throughout this contribution:

### Assumption A

*Given a complete and correct model of the evolutionary dynamics on a suitable constructed space* $X$, *there is an additive metric distance measure* $t$ *on* $X$ *that measures the cumulative change along each lineage.*

An immediate consequence is that phylogenetic relationships can be reconstructed unambiguously if $t$ is known. There is, of course, no reason to think that Assumption A holds in real life. In particular, it is certainly violated by all processes that lead to reticulate patterns in evolution, such as incomplete lineage sorting, horizontal gene transfer, and hybridization (Gontier 2015). The purpose of this contribution, therefore, is to ask how much (or how little) we need to know about the “true” metric *t* to be able to infer the correct phylogenetic tree $T$. More precisely, we investigate here the consequence of distorted distance measurements: Suppose that instead of $t$ we can infer from the data only a “deformed” dissimilarity measure $d=\zeta (t)$, where $\zeta$ is an unknown function about which only some qualitative features can be known. We then ask: How much information about $t$, and thus the underlying phylogenetic tree, does $d$ still convey?

## Distance-based phylogenetics

A map $d:X\times X\rightarrow {\mathbb {R}}_0^+$ is a *metric* if it satisfies, for all $x,y,z\in X$:

(M0) $$d(x,x)=0$$
(M1) If $$d(x,y)=0$$ then $$x=y$$.
(M2) $$d(x,y)=d(y,x)$$.
(M3) $$d(x,y)+d(y,z)\ge d(x,z)$$.

Distance measures can be used for clustering and thus serve as a means of extracting hierarchical, i.e., tree-like, structures on a set of data.

The basis of distance-based phylogenetic methods is *additive metrics*, i.e., metrics that are representations of edge-weighted trees. Consider a tree $T$ with leaf-set $X$ and a length function $\ell$ defined on the edges of $T$. Recall that every pair of leaves $x$ and $y$ is connected by a unique path $\mathbf{p }_{xy}$ in $T$. The length of this path, i.e., the sum of its edge lengths, defines the distance $d_T(x,y)$. Additive metrics are those that derive from a tree in this manner. A famous theorem (Buneman 1974; Cunningham 1978; Dobson 1974; Simões-Pereira 1969) shows that additive metrics are characterized by the *four-point condition*: A metric is additive if and only if for any four points $u,v,x,y\in X$ holds

(MA) $$d(u,v)+d(x,y) \le \max {\left\{ \begin{array}{ll} d(u,x)+d(v,y) \\ d(u,y)+d(v,x) \end{array}\right. }.$$

The appearance of additive metrics in evolutionary processes can be justified rigorously for specific models. For example, Markovian processes on strings of fixed length lead to distances that can be estimated directly from the data: Denoting by $c_{ab}(x,y)$ the fraction of characters in which $x$ has state $a$ and $y$ has state $b$, which for each pair ($x,y$) can be arranged in a matrix ${\mathbf {C}}(x,y) = \big (c_{a,b}(x,y)\big )_{a,b}$. Steel (1994) showed that (the expected values of) $d(x,y):= -\ln \vert \mathrm {det}({\mathbf {C}}(x,y)\vert$ form an additive metric. Well-known results from phylogenetic combinatorics show that given an additive metric, the tree $T$ and its edge lengths can be reconstructed readily, see, e.g., the work of Apresjan (1966), Imrich and Stockiĭ (1972), Buneman (1974), Dress (1984), Bandelt and Dress (1992), Dress et al. (2010a). The well-known *neighbor-joining* algorithm (Saitou and Nei 1987), a special case of a large class of agglomerative clustering algorithms, furthermore, solves this problem efficiently and was shown to always compute the correct tree when presented with an additive metric, see the survey by Gascuel and Steel (2006) and the references therein. Additivity of the underlying metric is also assumed in a recent generalization of phylogenetic trees that allows data points to appear not only as leaves but also as interior vertices of the reconstructed tree (Telles et al. 2013).

A stronger condition than additivity is *ultrametricity*, which is characterized by the strong triangle equation

(MU) $$d(x,z)\le \max \{d(x,y),d(y,z)\}.$$

Condition (MU) means that all triangles are “isosceles with a short base”, i.e., the length of two sides of the triangles is equal and the third one is at least not longer than these two. Ultrametrics appear in phylogenetics under the assumption of the strong clock hypothesis, i.e., constant evolutionary rates (Dress et al. 2007). Dating of the internal nodes (Britton et al. 2007) transforms an (additive) phylogeny into an ultrametric tree. Ultrametrics are a special case of additive metrics.

Real-life data sets, unfortunately, almost never satisfy the four-point condition. As a remedy, Sattah and Tversky (1977) and Fitch (1981) suggested to consider a “split relation” on pairs of objects, often referred to as *quadruples*, defined by

1 $$\begin{aligned} uv \Vert xy \,\iff \, d(u,v)+d(x,y) < {\left\{ \begin{array}{ll} d(u,x)+d(v,y) \\ d(u,y)+d(v,x) \end{array}\right. } \end{aligned}.$$

The relation $\Vert$ has been studied extensively and, under certain additional conditions, can provide sufficient information for reconstructing phylogenetic trees (Bandelt and Dress 1986) or at least phylogenetic networks (Bandelt and Dress 1992; Grünewald et al. 2009). The approximation of a given metric by additive metrics or ultrametrics given some measure of the goodness of fit has also received quite a bit of attention (Farach et al. 1996; Agarwala et al. 1998; Apostolico et al. 2013).

Here, we ask under which conditions distance data that may deviate from additivity in a systematic manner still yield a phylogenetically (more or less) correct relation $\Vert$. This is different from the inference problems mentioned above: Our task is not to minimize a uniform error functional but to deal with systematic distortions of the distance measurements. In order to formalize the problem setting, we assume that the evolutionary process under consideration (operating on a space $X$) generates an additive metric $t: X\times X\rightarrow {\mathbb {R}}_0^+$. The catch is that we have no knowledge of $X$ and we cannot directly access $t$. We can, however, obtain partial knowledge from representations. That is, there is a function $\varphi :X\rightarrow Y$. The construction of the representation in $Y$ depends on our theory of what is important about the evolving system. In molecular phylogenetics, $Y$ may be chosen to be a space of sequences. In classical, morphology-based phylogenetics, the elements of $Y$ are character-based descriptions of animals; attempts to use molecular structures for phylogenetic purposes might use RNA secondary structures or labeled graph representations of protein 3D structures; a historic linguist might choose word lists or grammatical features.

Once we have decided on representations, we can turn to measuring (dis)similarities between them. The concrete choice of a distance measure ${{\tilde{d}}}:Y\times Y\rightarrow {\mathbb {R}}_0^+$ of course again depends on the theoretical conception of the underlying evolutionary process. We can easily reinterpret ${\tilde{d}}$ as a distance measure on $X$ by setting

2 $$\begin{aligned} d(x,y) := {{\tilde{d}}}(\varphi (x),\varphi (y)) \end{aligned}.$$

It is easy to see that $d:X\times X\rightarrow {\mathbb {R}}$ is a metric whenever ${\tilde{d}}$ is a metric and $\varphi :X\rightarrow Y$ is injective, i.e., whenever our representation is good enough to distinguish objects in $X$. There is no *a priori* reason to make this assumption, however. Consider, for example, RNA secondary structures as a function of the primary sequences. This map is highly redundant (Schuster et al. 1994); for example, most tRNAs share the standard clover-leaf structure despite very different sequences and divergence times that pre-date the common ancestor of all extant life forms (Eigen et al. 1989); distances between secondary structures therefore do not reflect all evolutionary processes. Formally, $d$ is not a metric but only a pseudometric in this case: It does not satisfy axiom (M1) any longer. We will ignore this complication here and assume for simplicity that $d:X\times X\rightarrow R_0^+$ is a metric.

The metric $d$ is of interest for phylogenetic purposes if it quantifies evolutionary divergence in a meaningful way. That is, we are concerned with the information about the underlying additive metric $t$ that can be extracted from $d$. Without additional assumptions on the relationships between $t$ and $d$, however, nothing much can be said. At the very least, our representation $(Y,{\tilde{d}})$ should be good enough to recognize whether one of two objects $y$ or $z$ has diverged further from a given reference point $x$ than the other. Hence, we assume that for all $x,y,z\in X$:

(m0) $$t(x,y)<t(x,z)$$ implies $$d(x,y)<d(x,z)$$.

In the absence of at least this very weak form of monotonicity, we cannot really hope to recover information about $t$ from measuring $d$. To our knowledge, property (m0) has not received much attention in the past. The following, stronger condition, however, has been considered extensively:

1. $$t(x,y)<t(u,v)$$ implies $$d(x,y)<d(u,v)$$

for all $u,v,x,y\in X$. This property is known as *(strong) monotonicity* (Kruskal 1964) and lies at the heart of non-metric multi-dimensional scaling, a set of techniques that aim at approximating dissimilarity data by a Euclidean metric (Borg and Groenen 2005). A commonly used criterion is to minimize the violations of condition (m1). It is interesting to note in this context that, given any input metric $d$, there is a always a Euclidean metric $\delta$ that is connected with $d$ by strong monotonicity, provided the embedding space is of sufficiently high dimension (Agarwal et al. 2007). In our context, it will be interesting to investigate whether there is an analogous result for additive metrics.

If we insist, in addition, that ties are preserved, i.e., that $t(x,y)=t(u,v)$ is equivalent to $d(x,y)=d(u,v)$, then there exists an increasing function $\zeta :{\mathbb {R}}_0^+\rightarrow {\mathbb {R}}_0^+$ such that $d=\zeta (t)$. In the following, we will consider this (more restrictive) setting in some detail.

## Metric-preserving functions

### Definition 1

A function $\zeta :{\mathbb {R}}_0^+\rightarrow {\mathbb {R}}_0^+$ is *metric-preserving* if for every metric $t:X\times X\rightarrow {\mathbb {R}}_0^+$ the function $d=\zeta \circ t$ is also a metric on $X$.

Consider the following properties:

1. $\zeta (t)=0$ if and only if $t=0$ (amenable)
2. $$\zeta (t+u)\le \zeta (t)+\zeta (u)$$ (subadditive)
3. $\zeta$ is non-decreasing.

A theorem by Kelley (1955, p. 131) states that (Z1), (Z2), and (Z3) together are sufficient conditions for $\zeta$ to be metric-preserving. One can show, furthermore, that (Z1) and (Z2) are necessary (Corazza 1999). Property (Z3) is sufficient but not necessary, as shown by several examples of metric-preserving functions that fail to be non-decreasing (Doboš 1998; Corazza 1999). A necessary and sufficient condition (Wilson 1935; Borsik and Doboš 1981; Das 1989) is that $\zeta$ is amenable, (Z1), and satisfies

(Z*) $$\displaystyle \max _{w=|t-u|} \zeta (w) \le \zeta (t)+\zeta (u)$$.

It can also be shown that any concave amenable function is metric preserving (Doboš 1998). If $d=\zeta \circ t$ satisfies (m0), then (Z3) holds. We therefore restrict ourselves to amenable, subadditive, non-decreasing functions. Furthermore, we assume for convenience that $\zeta$ is continuous.

**Fig. 1:**
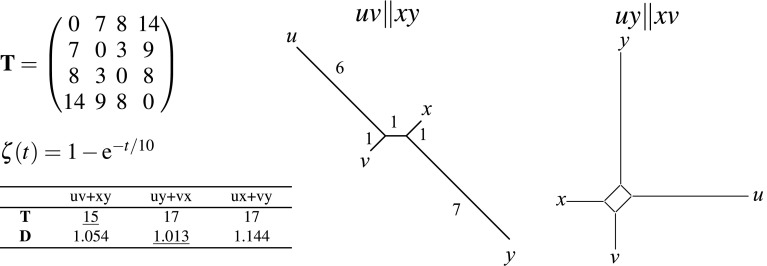
Metric-preserving transformations do not preserve the relation $\Vert$. The distance matrix ${\mathbf {T}}$ corresponds to the tree in the middle and, according to Eq. (1), satisfied $uv\Vert xy$. The function $\zeta$ satisfies (Z1), (Z2), (Z3) and is smooth. The transformed distance matrix ${\mathbf {D}}= \zeta ({\mathbf {T}})$ is presented by the networks shown on the r.h.s. (computed with SplitsTree (Huson and Bryant 2006). Here, $d(u,y)+d(x,v)$ is the distance pair with the shortest distance sum, i.e., it corresponds to the quadruple $uy\Vert xv$. This split corresponds to the longer one of the two side lengths of the box

We say that $\zeta$ is a.m.-preserving (ultrametric-preserving) if $\zeta \circ t$ is an additive metric whenever *t* is an additive metric (ultrametric). It was shown recently that a function $\zeta$ preserves ultrametricity if and only if it is amenable (Z1) and non-decreasing (Z3) (Pongsriiam and Termwuttipong 2014). In Appendix, we prove:

### Lemma 1

*If* $\zeta$ *is a.m.-preserving, then it is also ultrametric-preserving.*

This implies in particular that an a.m.-preserving function is non-decreasing. It will not come as a surprise that nonlinear distortions do not preserve additivity.

### Theorem 1

*If* $\zeta$ *is a.m.-preserving, then* $\zeta (t)=\alpha t + \beta$ *holds for all* $t>0$ *with* $\alpha ,\beta \ge 0$.

A proof can be found in Appendix. The importance of this theorem lies in the fact that any nonlinear distortion of the metric *t* necessarily destroys additivity and thus, depending on the algorithm employed, may result in the reconstruction of an incorrect phylogeny.

Given the importance of the relation $\Vert$, it is natural to ask whether—or under what conditions—at least this relation is preserved. The example in Fig. 1 shows, however, that the relation $\Vert$ is not necessarily preserved under transformations satisfying (Z1), (Z2), and (Z3). The example of Fig. 1 is reminiscent of the effect of *long branch attraction* (LBA) in parsimony-based methods (Felsenstein 1978; Bergsten 2005), which can also be understood the consequence of underestimating the impact of homoplasy, i.e., “back-mutations.”

## Multiple features

A reasonable approach to devise a distance measure for a set of objects is to use a representation in terms of a collection of features, i.e., to consider a product space $Y=\prod _i Y_i$ with distance measures ${\tilde{d}}_i: Y_i\times Y_i\rightarrow {\mathbb {R}}_0^+$ independently defined for each of the features. Each feature can be seen as an independent representation, $\varphi _i:X\rightarrow Y_i$, and thus, we may reinterpret the ${\tilde{d}}_i$ as different distance measures on *X*, i.e., $d_i:X\times X\rightarrow {\mathbb {R}}_0^+$ with $d_i(x,y):={\tilde{d}}_i(\varphi _i(x),\varphi _i(y))$. In this setting, it seems most natural to assume that $d_i$ is just a pseudometric.

It is well known that any nonnegative linear combination of pseudometric $d:=\sum _i a_i d_i$ with $a_i\ge 0$ is again a pseudometric. To avoid trivial cases, assume $a_i>0$. Then, $d$ is a metric whenever $x\ne y$ implies that there is a feature $i$ such that $d_i(x,y)>0$. The most general ways to combine metrics are given by the *generalized metric-preserving* transforms, i.e., functions $\xi :({\mathbb {R}}_0^+)^N \rightarrow {\mathbb {R}}_0^+$ with the property that $d=\xi (t_1,\dots ,t_n)$ is a metric whenever each $t_i$, $1\le i\le N$, is a metric (Das 1989). These functions have a characterization that naturally generalizes (Z1) and (Z*) to multiple arguments.

### Theorem 2

*If* $\xi :({\mathbb {R}}_0^+)^N \rightarrow {\mathbb {R}}_0^+$ *transforms additive metrics* $d_i$ *consistent with the same underlying tree* $T$ *into a metric* $\xi (d_1,\dots ,d_N)$ *that is again compatible with* $T$, *then* $\xi = \xi _L+\xi _D$ *where*

(i) $$\xi _L:({\mathbb {R}}_0^+)^N \rightarrow {\mathbb {R}}_0^+, (t_1,\dots ,t_N)\mapsto \sum _{i=1}^N a_i t_i$$ *with* $$a_i\ge 0$$,
(ii) $\xi _D$ *is a nonnegative linear combination* $(t_1,\dots ,t_N)\mapsto \sum _{i=1}^N b_i d^D_i$ where $d^D_i$ *is the standard discrete metric applied to the* $ith$ *component, i.e., the argument of* $t_i$.
(iii) *for each* $i$, *at least one of* $a_i$ *and* $b_i$ *is nonzero*.

### Proof

Suppose all component metrics $d_j$ are discrete except for $d_i$, $i\ne j$. Then, $d_i\mapsto \xi (d_1,\dots ,d_j,\dots , d_N)$ is linear with nonnegative slope for $d_i>0$ as an immediate consequence of Theorem 1, i.e., condition (i) is necessary. Theorem 1 furthermore implies that the contribution for each feature *i* is necessarily of the form $a_i t_i+b_i d^D_i$ with $a_i,b_i \ge 0$. To ensure that we have a metric, each constituent must be a metric, i.e., at least one of $a_i$ and $b_i$ must be nonzero. □

In essence, Theorem 1 characterizes the distance measures that are “good” for phylogenetic purposes: These exactly are the ones that are linear combinations of distance measures that themselves are additive. In particular, therefore, alignment-free phylogenetic methods are guaranteed to work only when their distance measure approximates an additive measure, or, equivalently, when they approximate a distance for which a transformation to an additive distance is known (and used for the phylogenetic reconstruction).

## Inferring $\zeta$ transformations

The theoretical considerations above lead to the conclusion that the key problem for phylogenetic inference from data without a completely understood underlying model is to find monotonic transformations that make the original data as additive as possible **before** applying distance-based phylogenetic methods. It is important to realize that this is **not** the same problem as extracting the additive part of a given metric using, e.g., split decomposition. To see this, consider the metric distance matrix

3 $$\begin{aligned} {\mathbf{D }} = \left( \begin{matrix} 0.000 &{} 0.503 &{} 0.551 &{} 0.753 \\ 0.503 &{} 0.000 &{} 0.259 &{} 0.593 \\ 0.551 &{} 0.259 &{} 0.000 &{} 0.551 \\ 0.753 &{} 0.593 &{} 0.551 &{} 0.000 \\ \end{matrix}\right) \end{aligned}.$$

The transformation $t = -10\ln (1-d)$ recovers the additive metric of Fig. 1 (up to small rounding errors) and thus recovers the tree in Fig. 1. Its split decomposition, on the other hand, yields the network on the r.h.s. of the figure with isolation indices $\alpha (xv|uy)=0.066$ and $\alpha (xy|uv)=0.045$. Any reasonable methods for fitting an additive tree thus will pick up the a quadruple with the $xv\Vert uy$ from these distances.

Consider now a function $\tau$ that, given a metric distance matrix ${\mathbf {D}}=(d(x,y))_{x,y}$ as input, produced a “best-fitting” additive metric distance matrix of the same dimension as output. More formally, denote by ${\mathbb {M}}_n$ the set of all metrics on *n* points, and let ${\mathbb {M}}=\bigcup _{n>1} {\mathbb {M}}_n$.

### Definition 2

A function $\tau : {\mathbb {M}}\rightarrow {\mathbb {M}}$ is *a.m.-consistent* if the following conditions are satisfied:

(i) If ${\mathbf {D}}\in {\mathbb {M}}_n,$ then $\tau ({\mathbf {D}})\in {\mathbb {M}}_n$ is an additive metric.
(ii) If ${\mathbf {D}}\in {\mathbb {M}}_n$ is an additive metric, then ${\mathbf {D}} = \tau ({\mathbf {D}})$.

The neighbor-joining algorithm (Saitou and Nei 1987) is a well-known example of an a.m.-consistent function $\tau$ (Gascuel and Steel 2006). Another example is the non-prime part of the split decomposition (Bandelt and Dress 1992). Given a distance matrix ${\mathbf {D}}$ and an a.m.-consistent function $\tau$, a natural measure for the deviation from additivity is $\vert {\mathbf {D}}-\tau ({\mathbf {D}})\vert$ with some matrix norm $\vert \,.\,\vert$. In particular, $\vert {\mathbf {D}}-\tau ({\mathbf {D}})\vert =0$ if and only if ${\mathbf {D}}$ is an additive metric.

Let us now return to Assumption A and characterize distances that derive from additive metrics in a simple manner:

### Lemma 2

*Let* ${\mathbf {D}}$ *be a metric distance matrix, let* $\tau$ *be an a.m.-consistent function, suppose* $\zeta$ *is invertible, increasing, and subadditive, and let* $\vert \,.\,\vert$ *be a matrix norm. Then, there is an additive distance matrix* ${\mathbf {T}}$ *with* ${\mathbf {D}}=\zeta ({\mathbf {T}})$ *if and only if* $\vert {\mathbf {D}} - \zeta (\tau (\zeta ^{-1}({\mathbf {D}})))\vert =0$.

### Proof

Invertibility of $\zeta$ implies that ${\mathbf {D}}=\zeta ({\mathbf {T}})$ is equivalent to ${\mathbf {T}}=\zeta ^{-1}({\mathbf {D}})$. Now ${\mathbf {T}}=\tau ({\mathbf {T}})=\tau (\zeta ^{-1}({\mathbf {D}}))$ if and only if ${\mathbf {T}}$ is additive. Using invertibility of $\zeta$ again, this is in turn equivalent to ${\mathbf {D}}=\zeta ({\mathbf {T}})=\zeta (\tau (\zeta ^{-1}({\mathbf {D}})))$. Since the matrix norm $\vert \,.\,\vert$ vanishes only for the 0-matrix, the Lemma follows. □

Lemma 2 immediately suggests to search for $\zeta$ by minimizing the error functional

4 $$\begin{aligned} {\varDelta }(\zeta ):= \vert {\mathbf {D}} - \zeta (\tau (\zeta ^{-1}({\mathbf {D}})))\vert \end{aligned}.$$

By Lemma 2, ${\mathbf {D}}$ derives from an additive metric if and only if a $\zeta$ with ${\varDelta }(\zeta )=0$ exists. Otherwise, we obtain an approximately additive source metric $\zeta ^{-1}({\mathbf {D}})$ that then serves as the best available input for phylogenetic reconstruction. In this case, the values of ${\varDelta }(\zeta )$ as well as the estimate $\zeta ^{-1}({\mathbf {D}})$ that is found by minimizing ${\varDelta }(\zeta )$ will in general depend on both the a.m.-consistent function $\tau$ and the matrix norm $\vert \,.\,\vert$.

**Fig. 2:**
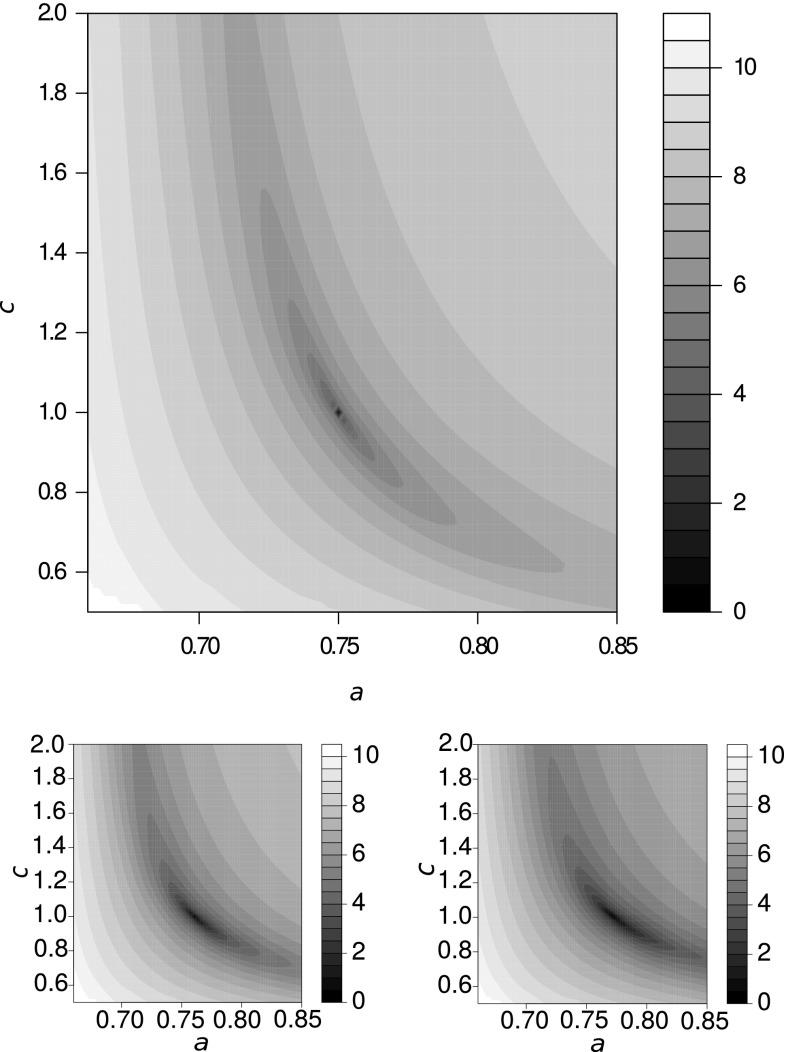
Empirical estimation of a transformation $\zeta$. Top: The relevant parameters $a$ and $c$ of the stretched exponential transform Eq. (5) can be estimated with the help of Eq. (4). Plotting ${\varDelta }(\zeta )$ as a function of the parameters $a$ and $c$ in Eq. (5) shows that the minimal discrepancy is indeed found at the theoretical values $a=3/4$ and $c=1$ used to generate the transformed distance matrix ${\mathbf {D}}$ corresponding to a tree with 100 leaves. The color scale on the r.h.s. of the panel refers to ln$(1+ {\varDelta }(\zeta ))$. Below: The two small panels show the effect of increasing levels of measurement noise (left: $\varepsilon =0.1$, right: $\varepsilon =0.2$, see “Appendix 2” for details)

As a proof of principle, we first produced an artificial distance matrix ${\mathbf {D}}$ by transforming distance of a randomly generated tree with 100 leaves using the Jukes–Kantor rule (Jukes and Cantor 1969) corresponding to a four-letter alphabet and scaling the mutation rate such that back-mutations play a role but distances are not completely saturated. We then make the assumption that the measured data might depend on the unknown additive scale via a stretched exponential transformation of the form

5 $$\begin{aligned} \zeta (t):=a(1-\exp (-b\,t^{c})) \end{aligned}$$

with unknown parameters $a$, $b$, and $c$. Figure 2(top) shows that the correct values of $a=3/4$ and $c=1$ can be inferred by using Eq. (4) to minimize the discrepancy ${\varDelta }(\zeta )$. In “Appendix 2,” we show more formally that the parameter $b$ is arbitrary and hence cannot be inferred. Intuitively, this follows from the fact that $b$ only scales the time axis and hence constitutes a purely additive transformation of the distance, which canceled in Eq. (4) by the application of $\zeta ^{-1}$.

Real-life distance data of course are not perfectly additive. We therefore simulated sequence data by introducing substitutions independently at each sequence position according to a first order Markov process along all edges of a given phylogenetic tree. In order to tune the level of noise, we considered different linear combinations of the theoretical and the simulated data, see “Appendix 2” for details. We found that the estimation of $\zeta$ via Eq. (4) works well for small levels of sampling noise. For large noise levels, however, there are systematic biases. These appear to depend strongly on the choice of the matrix norm $\vert \,.\,\vert$. Clearly, a better understanding of the numerical problems associated with this inference problem will be necessary before the conceptually simple workflow proposed here can be applied to real-life data.

## Discussion and conclusions

It has been realized already in the early days of computational phylogenetics that suitable transformation of distance data, e.g., using the Jukes–Cantor transformation, can increase the additivity and thus conceivably improve the quality of phylogenetic reconstructions (Vach 1992). A main insight in this contribution is that it is, at least in principle, possible to infer the correct distance transformation from the measured data only. As a consequence, the correct inference of phylogenetic relationships is possible not only for additive distances but also for the large class of distances that arise from additive metrics with a monotonic metric-preserving function.

At the same time, our results suggest that there are limits to phylogenetic inference. Whenever the available data cannot be transformed into an additive metric (at least approximately, i.e., up to measurement noise), there seems little hope to justify the interpretation of the results of hierarchical clustering (which of course can be performed on any kind of distance or similarity data) as a phylogeny. It is important to note, however, that our discussion has focused on metric-preserving functions, i.e., “uniform” transformations of the distance data. It is entirely possible to employ more general schemes that further extend the realm of phylogenetically meaningful data. For instance, the results of “Multiple features” section show that for data comprising multiple types of descriptors, distances extracted from the different subclasses *c* can be transformed with different functions $\zeta _c$. Such an approach might even be useful to distinguish phylogenetically informative from problematic classes of features.

On a more conceptual level, our results show that detailed mechanistic models of the underlying evolutionary process are **not logically necessary** for phylogenetic inference. It is, in fact, sufficient that the measured distance data can be transformed to an additive metric by means of a monotonic metric-preserving function. This is not to say that a mechanistic understanding of the process is not useful or desirable. After all, a mechanistic model will, at the very least, typically imply the functional form of the transformation function $\zeta$. The inference of $\zeta$ from real-world data remains an important open problem. The issue to be explored is not only the limiting effect of measurement noise and inherent deviations from additivity due to horizontal gene transfer, incomplete lineage sorting, etc., but also numerical issues such as the fact that, in large trees, a substantial fraction of all pairwise distances takes values very close to the diameter of the tree. This seems to cause a particular susceptibility to measurement noise. Systematic simulation studies well beyond the scope of this contribution will be required to address this issues.

A potential alternative to Eq. (4) is the minimization of some measure of tree-likeness for the transformed matrix $\zeta ^{-1}({\mathbf {D}})$. Attractive candidates are the corresponding parameters of statistical geometry (Eigen et al. 1988; Nieselt-Struwe 1997) and the related “$\delta$-plots” advocated by Holland et al. (2002). It is not obvious, however, how these measures react to the changes in scale invariably introduced by $\zeta$. This issue does not arise in the context of Eq. (4) because the effects cancel due to the appearance of both $\zeta ^{-1}$ and $\zeta$.

It is interesting to note that our results also provide an *a posteriori* explanation for the observation that alignment-free methods work best in phylogenetic applications when the distances correlate well with alignment-based distances (Haubold et al. 2009; Morgenstern et al. 2017; Thankachan et al. 2017). It will be interesting to see whether other types of distances, such as compression distances (Kocsor et al. 2006; Penner et al. 2011), admit a transformation that makes them approximately additive.

Finally, several mathematical questions arise naturally from the results presented here. First, we may ask whether it is possible to replace condition (m1) by weaker requirements, such as (m0)? Even more generally, to what extent can arbitrary rate variations be accommodated? We know of course that they are harmless in an underlying additive metric—but what is the most general distortion that can be accommodated? Complementarily, it will be of interest to characterize the functions that preserve circular (Kalmanson 1975) and weakly decomposable metrics (Bandelt and Dress 1992), respectively.

## Appendix 1: Proofs

### Proof of Lemma 1

Since every ultrametric is additive, an a.m.-preserving function must transform every ultrametric into an additive metric. Being a function, $\zeta$ in particular transforms isosceles triangles into isosceles triangles. In particular, it preserves equilateral triangles.

Consider the set of ultrametrics $q$ on 4 points satisfying $uv\Vert xy$. The four isosceles triangles are $u|xy, \, v|xy, \, x|uv, {\text{and}}\, y|uv$. Therefore, $q(u,x)=q(u,y)> q(x,y),$ $q(v,x)=q(v,y)>q(x,y),$ $q(x,u)=q(x,v)>q(u,v),$ and $q(y,u) = q(y, v) > q(u, v), i.e., c := q(u, x) = q(u, y) = q(x, v) = q(y, v)>\!;\, q(x, y), q(u, v).$ If the $\zeta$-transformed additive metric satisfies $uv\Vert _{\zeta } xy,$ then these four triangles still have short base. Recall that $q$ is an ultrametric if and only if every triangle is isosceles with short basis or equilateral. Therefore, $\zeta \circ q$ is again an ultrametric. Otherwise, suppose $ux \Vert _{\zeta } vy$ holds w.r.t. to the transformed metric. Then, additivity thus implies $\zeta (q(u,x))+\zeta (q(v,y)) < \zeta (q(u,v))+\zeta (q(x,y)) = \zeta (q(u,y))+\zeta (q(x,v)),$ i.e., $2\zeta (c)<2\zeta (c)$, a contradiction. The same result is obtained assuming $uy \Vert _{\zeta } vx$. In the degenerate case, no quadruple exists and thus $\zeta (q(u,v))+\zeta (q(x,y))=2\zeta (c)$. Since $q(u,v)$ and $q(x,y)$ can vary independently of each other, $\zeta$ must be constant, and thus, $\zeta \circ d$ is the trivial discrete metric, which is also an ultrametric. Hence, $\zeta$ is ultrametric-preserving on any subset of four points and thus in particular also preserves ultrametricity of all triangles. □

### Proof of Theorem 1

The discrete metric is additive; hence, any function $\zeta$ that is constant on ${\mathbb {R}}^+$ is a.m.-preserving. As a consequence of Lemma 1 and Pongsriiam and Termwuttipong 2014, we know that any a.m.-preserving function is amenable and non-decreasing. In the following, we therefore assume that $\zeta$ is amenable, not constant on ${\mathbb {R}}^+$, and non-decreasing.

Consider the set of additive metrics on four points satisfying $$t_{uv}+t_{xy}<t_{ux}+t_{vy}=t_{uy}+t_{vx}$$. Then, for some $$\epsilon$$ sufficiently small, the metric $$t'$$ defined by $$t'=t$$ except $$t'_{ux}=t_{ux}+\epsilon$$ and $$t'_{vx}=t_{vx}+\epsilon$$ is again an additive. Thus, $$\zeta (t_{ux}+\epsilon )-\zeta (t_{vx}+\epsilon )=t_{uy}-t_{vy}$$, a constant. It is easy to see that $$t_{vx}$$ and $$t_{ux}$$ can be chosen arbitrarily (first choose an isolation index $$\alpha$$ for $$uv\Vert xy$$ such that $$\alpha < \min (t_{vx},t_{ux})$$ and then pick $$t_{uv}$$ and $$t_{xy}$$ sufficiently small). Thus, for every $$a,b>0$$ and sufficiently small $$|\epsilon |$$, we have $$\zeta (a+\epsilon )=\zeta (b+\epsilon )+h_{ab}$$. Let us fix *a* and consider the partial function $$h_a: b\mapsto h_{ab}$$. Suppose $$h_a$$ is not constant. Then, then there is a point $$b'':=\inf \{b'>b| h_{ab}\ne h_{ab'}\}$$. Since we know that $$h_{a}$$ is constant in a neighborhood of $$b$$, we have $$b''>b$$. By construction $$h_{ab'}=h_{ab}$$ for all $$b'\in [b,b'')$$. But $$h_a$$ is also constant in an open neighborhood of $$b''$$, which has a non-empty intersection with $$[b,b'')$$. Thus, $$h_{ab}=h_{ab''}$$, a contradiction. Renaming the arguments, there is a function $$h$$ such that

6 $$\begin{aligned} \zeta (x+a)-\zeta (x)=h(a) \end{aligned}$$

for all $a>0$ and $x>0$.

Replacing $a$ by $pa$ for $p\in {\mathbb {N}}$ yields $\zeta (x+pa)-\zeta (x)=h(pa)$, while substituting $x$ with $x+(p-1)a$ yields $\zeta (x+pa)-\zeta (x+(p-1)a)=h(a)$. Substituting $p$ by $p-1$ and adding the resulting equation lead to $\zeta (x+pa)-\zeta (x+(p-2)a)=2h(a)$ and thus eventually $\zeta (x+pa)-\zeta (x)=ph(a)$. Taken together, we have $h(pa)=ph(a)$. Replacing $a$ by $a$ / $p$ shows $h(a)=ph(a/p)$ and thus $p'h(a)=ph(p'a/p)$ for all $p,p'\in {\mathbb {N}}$. That is, $h(pa)=ph(a)$ for all $p\in {\mathbb {Q}}$. Since $\zeta$ is non-decreasing, we see that $a\mapsto h(a)=\zeta (x+a)-\zeta (x)$ is also non-decreasing. Therefore, $p'h(a)\le h(pa) \le p''h(a)$ holds for all $p\in {\mathbb {R}}$ and all $p',p''\in {\mathbb {Q}}$ with $p'\le p\le p''$. Using the well-known fact that ${\mathbb {Q}}$ is dense in ${\mathbb {R}}$ conclude that $h(pa)=p h(a)$ holds for all $p\in {\mathbb {R}}$. In particular, we have $h(a)=ah(1)$. Substituting this into Eq. (6) and setting $x=1$ yield $\zeta (a+1)-\zeta (1)=a h(1)$. Setting $x=a+1$ and rearranging the terms, finally, yield

7 $$\begin{aligned} \zeta (x)= h(1)x + (\zeta (1)-h(1)) \end{aligned}$$

for all $x>0$. The theorem now follows by observing that both the slope *h*(1) and the intercept $\zeta (1)-h(1)$ must be nonnegative since $\zeta$ is amenable and non-decreasing. □

## Appendix 2: On the example of Fig. 2

In Fig. 2, we considered distance data generated from an additive tree using a transformation of the form Eq. (5), which has the inverse $\zeta ^{-1}(u) = \left( -(1/b)\ln (1-u/a)\right) ^{1/c}$, with parameters $a$, $b$, $c$ fixed a some values $a_0$, $b_0$, $c_0$, which we pretend not to know. Transforming them with $\zeta ^{-1}$ with the correct value $a_0$ but arbitrary choices of $b$ and $c$ yields transformed distances

8 $$\begin{aligned} q=\zeta ^{-1}_{b,c}(\zeta _{b_0,c_0}(t)) = \root c \of {\frac{b_0}{b}}\, t^{c_0/c} \end{aligned}.$$

The coefficients $b_0$ and *b* appear only in the multiplicative factor $(b_0/b)^{1/c},$ and this does not affect additivity of the metric because the function $\tau$ must satisfy $\tau (\alpha {\mathbf {T}}) = \alpha \tau ({\mathbf {T}})$ for input matrices close to ${\mathbf {T}}$ that are almost additive. It follows that the scaling factor $b_0$ of the time axis cannot be inferred by minimizing the discrepancy in Eq. (4). This does not matter for phylogenetic reconstruction, however, because the scaled distance matrix $\alpha {\mathbf {T}}$ corresponds to the same phylogenetic tree as ${\mathbf {T}}$. In contrast, choosing an exponent $c\ne c_0$ causes a nonlinear distortion and thus causes a nonzero discrepancy in data. It is also easy to see that any choice of $a\ne a_0$ also causes a nonzero discrepancy, and hence, $a$ can be inferred.

In order to construct a data set with tunable levels of sampling error, we used the tree ${\mathbf {T}}$ as “scaffold” to simulate the evolution of four-letter sequences of length $N=10000$ for 100 time units with an per site substitution rate of $\mu =0.007$. Denote by ${\mathbf {D}}_H$ the empirically determined scaled Hamming distances for a particular instance of the simulated sequences. By construction, the expected distance matrix for this model is ${\mathbf {D}}^* = \zeta ({\mathbf {T}})$ with $a=3/4$ and $c=1$. Hence, the sampling variance can be tuned by using linear combinations of ${\mathbf {D}}^*$ and ${\mathbf {D}}_H$. We used convex combinations of the form ${\mathbf {D}}=(1-\epsilon ){\mathbf {D}}^* + \epsilon {\mathbf {D}}_H$. Note that the limit $\epsilon \rightarrow 0$ corresponds to sequences of infinite length, which allow an arbitrarily accurate estimation of the expected distances.
